# Delirium diagnosis defined by cluster analysis of symptoms versus diagnosis by DSM and ICD criteria: diagnostic accuracy study

**DOI:** 10.1186/s12888-016-0878-6

**Published:** 2016-05-26

**Authors:** Esteban Sepulveda, José G. Franco, Paula T. Trzepacz, Ana M. Gaviria, David J. Meagher, José Palma, Eva Viñuelas, Imma Grau, Elisabet Vilella, Joan de Pablo

**Affiliations:** Hospital Psiquiatric Universitari Institut Pere Mata, IISPV, Universitat Rovira i Virgili, Centro de Investigación Biomédica en Red de Salud Mental (CIBERSAM), Reus Tarragona, Spain; Grupo de Investigación en Psiquiatría de Enlace, Escuela de Ciencias de la Salud, Facultad de Medicina, Universidad Pontificia Bolivariana, Medellín, Colombia; Indiana University School of Medicine, Indianapolis, IN USA; Tufts University Medical Shool, Boston, MA USA; Universidad San Buenaventura, Faculty of Psychology, Medellín, Antioquia Colombia; Department of Psychiatry, University of Limerick School of Medicine, Limerick, Ireland; School of Medicine, University College Dublin, Dublin, Ireland

**Keywords:** Delirium, Dementia, Delirium rating scale-revised-98, Sensitivity and specificity, Reliability, Diagnostic and statistical manual of mental disorders, International classification of diseases, Cluster analysis, Discriminant analysis

## Abstract

**Background:**

Information on validity and reliability of delirium criteria is necessary for clinicians, researchers, and further developments of DSM or ICD. We compare four DSM and ICD delirium diagnostic criteria versions, which were developed by consensus of experts, with a phenomenology-based natural diagnosis delineated using cluster analysis of delirium features in a sample with a high prevalence of dementia. We also measured inter-rater reliability of each system when applied by two evaluators from distinct disciplines.

**Methods:**

Cross-sectional analysis of 200 consecutive patients admitted to a skilled nursing facility, independently assessed within 24–48 h after admission with the Delirium Rating Scale-Revised-98 (DRS-R98) and for DSM-III-R, DSM-IV, DSM-5, and ICD-10 criteria for delirium. Cluster analysis (CA) delineated natural delirium and nondelirium reference groups using DRS-R98 items and then diagnostic systems’ performance were evaluated against the CA-defined groups using logistic regression and crosstabs for discriminant analysis (sensitivity, specificity, percentage of subjects correctly classified by each diagnostic system and their individual criteria, and performance for each system when excluding each individual criterion are reported). Kappa Index (K) was used to report inter-rater reliability for delirium diagnostic systems and their individual criteria.

**Results:**

117 (58.5 %) patients had preexisting dementia according to the Informant Questionnaire on Cognitive Decline in the Elderly. CA delineated 49 delirium subjects and 151 nondelirium. Against these CA groups, delirium diagnosis accuracy was highest using DSM-III-R (87.5 %) followed closely by DSM-IV (86.0 %), ICD-10 (85.5 %) and DSM-5 (84.5 %). ICD-10 had the highest specificity (96.0 %) but lowest sensitivity (53.1 %). DSM-III-R had the best sensitivity (81.6 %) and the best sensitivity-specificity balance. DSM-5 had the highest inter-rater reliability (K =0.73) while DSM-III-R criteria were the least reliable.

**Conclusions:**

Using our CA-defined, phenomenologically-based delirium designations as the reference standard, we found performance discordance among four diagnostic systems when tested in subjects where comorbid dementia was prevalent. The most complex diagnostic systems have higher accuracy and the newer DSM-5 have higher reliability. Our novel phenomenological approach to designing a delirium reference standard may be preferred to guide revisions of diagnostic systems in the future.

**Electronic supplementary material:**

The online version of this article (doi:10.1186/s12888-016-0878-6) contains supplementary material, which is available to authorized users.

## Background

Valid and reliable diagnostic criteria in order to correctly classify delirium are fundamental to guide identification, management and prognosis [1]. Validity of a test or set of criteria involves accuracy, determined in part through sensitivity and specificity, and usually measured against a “gold standard” that is considered valid.

Without an easily measured biological marker for delirium, its diagnostic criteria are the only gold standard for clinical diagnosis. Criteria have been evolving through iterations since the 1960’s. However, the use of criteria largely relying on experts’ consensus and epidemiological research can be circular [2–4]. Further, iterations of diagnostic classification systems may result in different delirium diagnosis status in the same patient population.

Cole et al. [5] reported diagnostic accuracies for DSM-III, DSM-III-R, DSM-IV, and ICD-10 delirium criteria using latent class analysis (a latent variable model to delineate latent discrete variables from observed discrete criteria that allow describing accuracy among them). They found a relatively low sensitivity for ICD-10, low specificity for DSM-IV and high sensitivity and specificity for the DSM-III-R criteria. Those subjects were assessed with DSM-III-R delirium criteria, Confusion Assessment Method (CAM), and Delirium Index, without mention about how other diagnostic criteria were evaluated or if they were imputed from the available data obtained with the instruments of the studies. Meagher et al. [6] compared performance of DSM-5 criteria, imputed using symptom ratings from the Delirium Rating Scale-Revised-98 (DRS-R98) items, against DSM-IV criteria as directly assessed in patients in their pooled database. They reported 30.0 % sensitivity and 99.0 % specificity for DSM-5 criteria using a “strict” approach while a “relaxed” interpretation performed more similarly to DSM-IV with 89.0 % sensitivity and 96.0 % specificity. Concordance was only 53.0 % for these approaches where “strict” DSM-5 appeared to be only delineating full syndromal delirium whereas DSM-IV detected milder cases as well. Therefore, it remains unclear which is the most useful diagnostic system.

An alternative method is to use an “agnostic” approach to categorizing delirium based on its features. Cluster analysis is a multivariate statistical method that identifies groups of cases according to similarity on certain well-accepted characteristics (phenotype) of a specific disorder [7] without the constraint of an a priori diagnostic system. Cluster analysis should be performed in populations with a wide range of diagnostic severity and complexity. The complexity of delirium detection increases when it occurs in the context of other neuropsychiatric disorders, especially dementia [8, 9].

The DRS-R98 is an ideal tool to evaluate the delirium phenotype because it was developed based on delirium symptom characteristics rather than any particular (a priori) diagnostic system [10]. It is a widely employed instrument for standardized evaluation of delirium phenomenology and has been revalidated in diverse countries across different clinical settings [10–18]. It was designed to evaluate the breadth and severity of known delirium characteristics and enabled delineation of its three core domains (cognitive, circadian, higher order thinking), its noncore aspects [19, 20], cognitive alterations [21, 22], motor subtypes [23, 24], subsyndromal phenotype [25–27] and longitudinal course of episodes [28–30]. It had high accuracy and nearly the same delirium diagnosis cut-off across diagnostic criteria (14.5 for DSM-III-R, DSM-IV, DSM-5 and 15.5 for ICD-10) in a sample with high prevalence of dementia [31], with very high inter-rater reliability (intra-class correlation coefficient >0.9 replicated in validation studies).

Conversely, studies of inter-rater reliability for delirium diagnostic criteria show more variable levels of agreement. Cameron et al. [32] reported a Kappa Index (K) of 0.62 for test-retest reliability of DSM-III in acute medical inpatients. Silver et al. [33] found an excellent inter-rater reliability for DSM-IV in critically ill pediatric patients (K =0.9). Malt et al. [34] evaluated ICD-10 in a general hospital via evaluation of written history cases by diverse clinicians and K for delirium diagnosis of about 50.0 %.

According to Kendler [35], a defining feature of mature sciences is their cumulative nature and its capacity to build on what has gone before. In this sense, evolution of diverse psychiatric criteria could be understood as an iterative process that should eventually increase accuracy and reliability of clinical diagnosis, though to measure the components of a condition, an independent way needs to be employed in order to avoid the presumption of truth of any classification system. We aimed to assess the accuracy of several diagnostic systems for delirium when tested against delirium and nondelirium reference groups defined in an “agnostic” fashion through cluster analysis of DRS-R98 items. To increase complexity our population had high dementia prevalence. We also measured inter-rater reliability of each system when applied by two evaluators from distinct disciplines.

## Methods

### Subjects

This is a cross-sectional prospective study of 200 consecutive patients admitted to a skilled nursing facility (Centro Sociosanitario Monterols, Tarragona, Spain). Patients were admitted from home, general hospital, assisted living or senior community for convalescence of medical-surgical conditions or control of geriatric conditions. Exclusion criteria were refusal to participate, coma/sedation, severe language disorder, or inability to speak Spanish.

### Ethics, consent and permissions

This study was performed in accordance to Declaration of Helsinki and approved by the Hospital Universitari de Sant Joan Ethics Committee (our corresponding evaluation center). All patients or their proxy, when Mini Mental State Examination (MMSE) score was <24 (taken as part of the initial evaluation at admission), gave their written consent to participate.

### Measures and instruments

Demographical and clinical data, including age, sex, marital and occupational status and years of education were collected. We also reviewed medical records for a recent diagnosis of delirium.

#### Charlson Comorbidity Index (Short form; CCI-SF)

Developed from the CCI with similar prognostic value [36], this version is based on history of 8 medical conditions: cerebrovascular accident, diabetes mellitus, chronic obstructive pulmonary disease, congestive heart failure, dementia, peripheral arterial disease, chronic renal failure and cancer, scored so that the first six receive 1 point and the last two receive 2 points. A CCI-SF score of 0 or 1 indicates no comorbidity, 2 low comorbidity, and ≥3 high comorbidity.

#### Spanish-Informant Questionnaire on Cognitive Decline in the Elderly (S-IQCODE)

Structured interview composed by 26 questions about cognitive and functional aspects of the patient during the last 5 years [37]. It is a valid approach to detect a probable dementia. Scores range from 26 to 130. We used the validated Spanish version with the recommended cut-off >85 for possible dementia [38].

#### Delirium Rating Scale Revised-98 (DRS-R98)

The DRS-R98 has descriptive anchors for rating the severity levels for each of its items (0 is normal to a maximum of 3) with a maximum scale score of 46 points. It measures severity of many delirium symptoms using phenomenologically anchored descriptions for item ratings and can also diagnose delirium. Its 16 items include 3 diagnostic items comprising the DRS-R98 Total scale where 13/16 items constitute the DRS-R98 Severity scale. The DRS-R98 measures core symptoms representing the 3 core domains of delirium (cognitive, circadian, higher order thinking) and noncore symptoms (psychotic and affective). It was originally validated using raters blinded to the diagnoses in five diagnostic groups of inpatients [10]. It has been subsequently translated and revalidated in countries outside of the U.S. The appropriate Spanish version was used [11], and the expert rater had ample experience in using the scale in delirium phenomenology studies. The Spanish DRS-R98 had very high inter-rater reliability (intraclass correlation coefficient >0.9 in both Colombian and Spanish samples) [11, 14], and excellent validity as shown by the area under the curve >0.9 (Receiver-Operator Characteristic analyses) when discriminating DSM-III-R, DSM-IV, DSM-5 or ICD-10 delirium in a sample of patients from the same facility of this study [31]. The DRS-R98 has been assessed against other neuropsychiatric disorders making it an ideal instrument to assess phenomenology [8, 10].

#### Clinical diagnostic criteria

We used four classification systems: the DSM-5, DSM-IV and DSM-III-R editions [39–41] and the ICD-10 for research [42]. We designed a diagnostic criteria checklist to systematically rate each item for all diagnostic criteria as present or not in order to ensure their complete evaluation.

### Procedures

After running a pilot test with 10 patients (not included in the study sample) to evaluate logistic difficulties and possible problems in using research instruments, all patients admitted to the facility were rated by three researchers from 24 to 48 h after admission (all evaluations were done within the same 24-h period). Researchers #1 (psychiatrist trained and experienced in delirium and dementia clinical and research evaluations) and #2 (neuropsychologist experienced in evaluation of delirium and dementia for research purposes) evaluated symptoms for the delirium diagnostic criteria checklist. Researcher #3, a psychiatrist experienced in delirium and dementia research, teaching, clinical assessment, and specifically trained on the DRS-R98, administered the Spanish DRS-R98. Evaluations were made independently by each researcher. Ratings were based on the previous 24 h period. Researcher #3 also compiled demographic and clinical information for this report and researchers #1 and #2 contacted the family or caregiver to obtain the S-IQCODE score. All of them had unlimited access to medical/nursing records or reports of any kind and to interview caregivers, and were blinded to information from each other.

### Statistical analysis and delineation of study groups

Data were analyzed using SPSS Statistics 17.0 and a spreadsheet.

Continuous variables are expressed as means ± standard deviation (SD). Chi-square test was used to compare categorical variables (continuity correction was used when appropriate) and *t* test for continuous ones. Statistical significance was set at *p* < 0.05.

#### Delineation of study groups without a priori criteria using cluster analysis of the DRS-R98

We analyzed DRS-R98 Severity Scale (items 1 to 13) using two-step cluster analysis with Log-likelihood as a measure of “distance” between item scores. This is an exploratory technique that reveals natural groupings within a set of data. It allowed us to automatically calculate the number of natural clusters within the dataset without any a priori specification of what that number should be. Schwarz’s Bayesian Criterion method was used for clustering (to avoid overfitting of the obtained clusters due to the high number of items). Before cluster analysis, we excluded possible colinearity issues by means of a principal components analysis of the items, where any *Eigenvalue* (i.e., the part of the total variance induced by a factor) close to zero suggests a colinearity problem. We used the Belsley criterion to define “close to zero”: values between 30 and 100 for the square root of the ratio between the higher and the lower *Eigenvalue* indicate moderate to strong colinearity problems. We did not find concerning colinearity because the higher *Eigenvalue* was 6.045 and the lower was 0.195 (square root of the ratio =5.567).

#### Discriminant analysis of DSM and ICD criteria for delirium over study groups

Logistic regressions and crosstabs were used to assess sensitivity, specificity, and percentage of subjects correctly classified by each diagnostic system and their individual criteria, and the corresponding 95.0 % confidence intervals (95 % CI) are reported. Values are also given for diagnostic systems when each of their individual criteria were excluded. Wald test p value was utilized to define if classification performance percentages against reference groups were significant. All discriminant analyses are for the performance of all diagnostic criteria assessed by Researcher #1 (psychiatrist) against DRS-R98 evaluation from Researcher #3 (psychiatrist). Frequency (percentage) of subjects positive for delirium according to each diagnostic system and for presence of their individual criteria was also assessed.

#### Inter-rater reliability of DSM and ICD criteria for delirium

We report Kappa Index (K) with its 95 % CI and Standard Error (SE) as measure of reliability of all diagnostic criteria and items (for all diagnostic criteria assessed by Researcher #1 vs. Researcher #2). K for diagnostic systems when each of their individual criteria (items) were excluded is reported also. Every K was interpreted according to the following ranges: <0.20 = unacceptable, 0.20–0.39 = questionable, 0.4–0.59 = acceptable, 0.60–0.79 = good, and 0.80–1 = excellent.

## Results

**Fig. 1:**
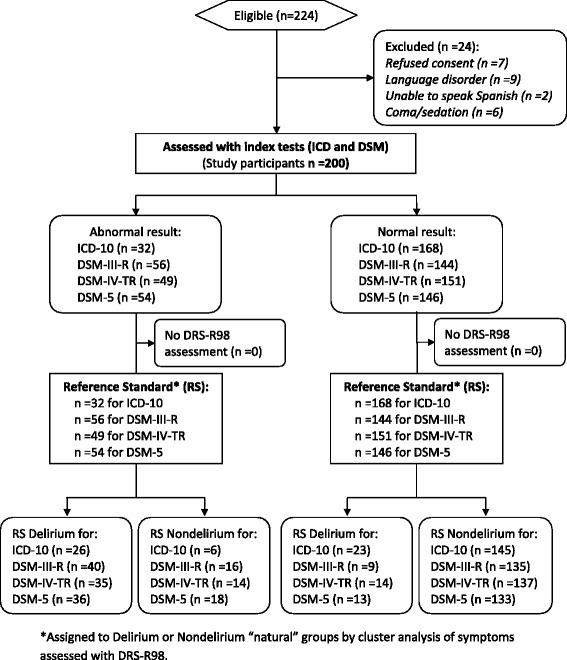
Flow diagram of participants. Delirium defined by cluster analysis of symptoms vs. diagnosis by DSM and ICD criteria in a sample with high prevalence of dementia

Figure 1 shows patients flow throughout the study. A total of 224 patients were admitted during the 14 months of patient collection. Reasons for exclusion were denied consent (*n* = 7), severe language disorder (*n* = 9), coma/sedation (*n* = 6), unable to speak Spanish (*n* = 2), leaving 200 who were included for analyses. Of these, the mean age was 78.3 ± 9.9 and 51.5 % were women.

### Groups defined according to cluster analysis

**Fig. 2:**
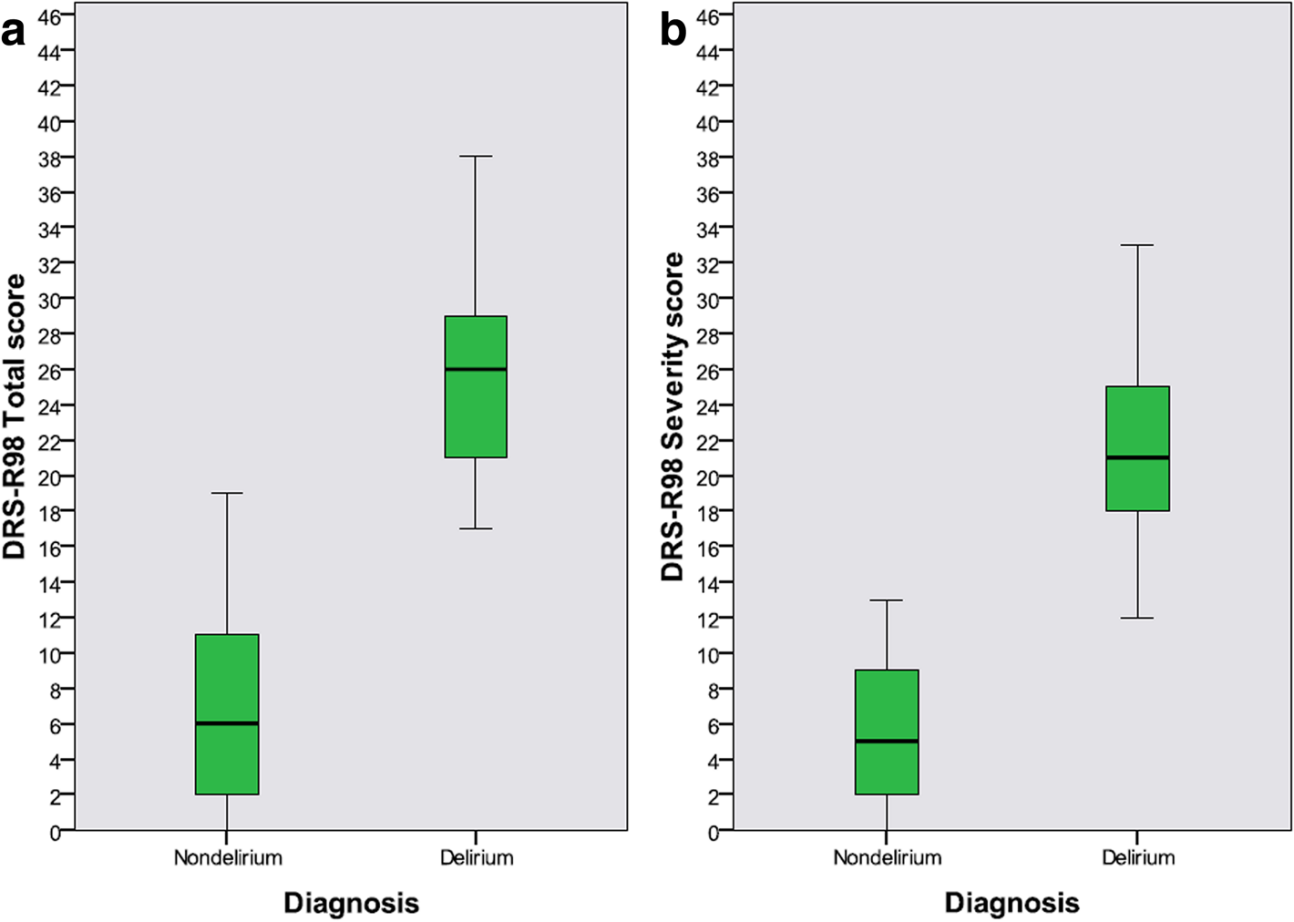
Study groups. Boxplots of DRS-R98 to illustrate the two study groups obtained using two-step cluster analysis. Part **a** shows distribution of DRS-R98 Total score for the delirium cluster (*n* = 49) and for the nondelirium cluster (*n* = 151). Part **b** shows DRS-R98 Severity score distribution for the same groups. Solid lines within boxes are median scores; boxes correspond to the middle 50.0 % of scores; tails indicate 25thpercentiles

Cluster analysis of DRS-R98 item scores resulted in a 2-natural cluster (or group) solution (nondelirium *n* = 151, delirium *n* = 49) (Fig. 2 boxplots). In nondelirium, the mean score for DRS-R98 Total was 6.67 ± 5.00 (range 0–19) and DRS-R98 Severity was 5.60 ± 3.82 (range 0–13). In delirium, the mean score for DRS-R98 Total was 25.59 ± 4.90 (range 17–38) and DRS-R98 Severity 21.29 ± 4.50 (range 12–33). There was minimal overlap between clusters except for small portions of their tails. Medians were also significantly different (median test *p* < 0.001).

### Population characteristics

Table 1 shows characteristics of the sample, divided into delirium and nondelirium groups using cluster analysis-defined groupings. The delirium group was older, had greater frequency of systemic infection as main diagnosis and a higher frequency of dementia as an antecedent. In both the whole sample and subsample of 117 with dementia (58.5 %), delirium subjects were more likely to have a comorbid diagnosis of dementia, and were more often on treatment with atypical antipsychotics. A past history of delirium was also more common in those with delirium.

**Table 1:** Demographic and clinical characteristics of the sample according to cluster analysis-defined delirium and nondelirium status

Variable	Whole sample		Dementia Subsample (S-IQCODE >85)	
	Nondelirium (*n* = 151)	Delirium (*n* = 49)	Nondelirium (*n* = 76)	Delirium (*n* = 41)
Age (years)	**77.46 ± 10.30**	**81.06 ± 8.08**	79.62 ± 7.48	81.12 ± 8.22
Education (years)	5.14 ± 4.21	4.61 ± 3.55	3.42 ± 3.32	4.29 ± 3.64
Charlson comorbidity score	1.81 ± 1.54	2.18 ± 1.18	2.07 ± 1.56	2.24 ± 1.18
Sex (%):				
Men	68 (45.0)	29 (59.2)	**26 (34.2)**	**26 (63.4)**
Women	83 (55.0)	20 (40.8)	**50 (65.8)**	**15 (36.6)**
Occupational status (%)				
Employed / Homemaker	6 (4.0)	2 (4.1)	2 (2.6)	1 (2.4)
Retired / Pensioner	143 (94.7)	47 (95.9)	74 (97.4)	40 (97.6)
Unemployed	2 (1.3)	-	-	-
Possible dementia^1^ (%)	**76 (50.3)**	**41 (83.7)**	N/A	N/A
Medications used^2^ (%):				
Anticholinergics	60 (39.7)	23 (46.9)	30 (39.5)	20 (48.8)
Typical antipsychotics	7 (4.6)	5 (10.2)	4 (5.3)	3 (7.3)
Atypical antipsychotics	**45 (29.8)**	**36 (73.5)**	**29 (38.2)**	**32 (78.0)**
Benzodiazepines	64 (42.4)	20 (40.8)	38 (50.0)	15 (36.6)
Cognitive enhancers	10 (6.6)	5 (10.2)	9 (11.8)	5 (12.2)
Five most common main diagnoses on admission (%)				
Dementia	**14 (9.3)**	**17 (34.7)**	**14 (18.4)**	**17 (41.5)**
Convalescence for fracture:				
Hip / Femur fracture	31 (20.5)	5 (10.2)	15 (19.7)	4 (9.8)
Other types	19 (12.6)	3 (6.1)	7 (9.2)	1 (2.4)
Psychiatric diagnosis	**17 (11.3)**	**-**	**11 (14.5)**	**-**
Cerebrovascular disease	15 (9.9)	7 (14.3)	7 (9.2)	5 (12.2)
Systemic infection	**9 (6.0)**	**8 (16.3)**	5 (6.6)	8 (19.5)
Previous diagnosis of delirium^3^	**18 (11.9)**	**15 (30.6)**	**12 (15.8)**	**13 (31.7)**
DRS-R98 Severity Score	**5.60 ± 3.82**	**21.29 ± 4.50**	**7.47 ± 3.30**	**21.63 ± 4.51**
DRS-R98 Total Score	**6.67 ± 5.00**	**25.99 ± 4.90**	**8.87 ± 4.37**	**25.76 ± 5.00**
DSM-III-R diagnoses (%)	**16 (10.6)**	**40 (81.6)**	**10 (13.1)**	**32 (78.0)**
DSM-IV diagnoses (%)	**14 (9.3)**	**35 (71.4)**	**7 (9.2)**	**27 (65.8)**
DSM-5 diagnoses (%)	**18 (11.9)**	**36 (73.5)**	**10 (13.1)**	**28 (68.3)**
ICD-10 diagnoses (%)	**6 (4.0)**	**26 (53.1)**	**5 (6.6)**	**20 (48.8)**

Data are shown as means ± SD unless denoted by frequencies, which are expressed as n (%). Bolded values reached significance at *p* < 0.05 for differences between delirium and nondelirium groups

*N/A* Not Applicable

^1^Based on S-IQCODE >85

^2^During 24 h before research evaluation

^3^As reported in clinical records

Delirium and nondelirium cases are listed according to the four diagnostic systems. The higher frequency was for DSM-III-R delirium whit 56/200 cases (28.0 %), and the lower was for ICD-10 with 32/200 cases (16.0 %); DSM-III-R delirium achieved the higher coincidence percentage with the reference standard delirium, ICD-10 obtained the lower (Table 1). Delirium was significantly more prevalent in the 117 with dementia than in the 83 without dementia for almost all diagnostic criteria: 8.4 % delirium in nondementia vs. 21.4 % in dementia subjects for ICD-10 (χ^2^ = 6.043, *p* = 0.014); 19.3 % vs. 32.5 % for DSM-5 (χ^2^ = 4.293, *p* = 0.038) and 16.9 % vs. 35.9 % for DSM-III-R (χ^2^ = 8.722, *p* = 0.003). There was a similar trend for DSM-IV, with 18.1 % vs. 29.1 % (χ^2^ = 3.169, *p* = 0.075).

### Criteria systems accuracy

Delirium classification performance characteristics for each diagnostic system and their individual criteria are shown in Table 2. All diagnostic systems correctly classified subjects similarly enough to the cluster-defined groups to be significant (Wald statistic *p* < 0.05). In the whole sample all diagnostic systems had very good accuracy, where the highest percentage of correctly classified cases was obtained by DSM-III-R criteria (87.5 %) and followed closely by DSM-IV (86.0 %), ICD-10 (85.5 %) and DSM-5 (84.5 %). The pattern was for all to have lower sensitivity than specificity especially evident for ICD-10 with specificity of 96.0 % and the lowest sensitivity of 53.1 %. In contrast, DSM-III-R had the best sensitivity (81.6 %) and the most balanced sensitivity-specificity values.

**Table 2:** Classification performance for delirium diagnostic systems and their individual criteria as compared to cluster analysis-defined groups

Classification Systems and their Criteria	Whole sample (*n* = 200)						Dementia subsample (*n* = 117)					
	Sensitivity %	95 % CI	Specificity %	95 % CI	Accuracy %	95 % CI	Sensitivity %	95 % CI	Specificity %	95 % CI	Accuracy %	95 % CI
DSM-III-R	81.6	67.5–90.8	89.4	83.1–93.6	**87.5**	81.9–91.6	78.0	62.0–88.9	86.8	76.7–93.2	**83.8**	75.5–89.7
A Alteration to maintain and shift attention	85.7 [81.6]	72.1–93.6	84.1 [89.4]	77.1–89.4	**84.5 [87.5]**	78.6–89.1	82.9 [78.0]	67.3–92.3	78.9 [86.8]	67.8–87.1	**80.3 [83.8]**	71.8–86.9
B Disorganized thinking	89.8 [81.6]	77.0–96.2	79.5 [87.4]	72.0–85.4	**82.0 [86.0]**	75.8–86.9	87.8 [78.0]	73.0–95.4	68.4 [85.5]	**56.6–78.3**	**75.2 [82.9]**	66.2–82.5
C Alterations in two of: consciousness, perception, sleep – wake cycle, motor activity, orientation and memory.	100 [81.6]	90.9–99.8	43.7 [89.4]	35.7–52.0	57.5 **[87.5]**	50.3–64.4	100 [78.0]	89.3–99.8	18.4 [86.8]	10.8–29.3	47.0 **[83.8]**	37.8–56.4
D Acute onset and fluctuation tendency.	81.6 [83.7]	67.5–90.8	87.4 [88.1]	80.8–92.1	**86.0 [87.0]**	80.2–90.3	78.0 [80.5]	62.0–88.9	85.5 [84.2]	75.1–92.2	**82.9 [82.9]**	74.6–89.0
E Evidenced or presumed etiological cause.	83.7 [81.6]	69.8–92.2	80.8 [89.4]	73.4–86.6	**81.5 [87.5]**	75.3–86.5	80.5 [78.0]	64.6–90.6	78.9 [86.8]	67.8–87.1	**79.5 [83.8]**	70.8–86.2
DSM-IV	71.4	56.5–83.0	90.7	84.6–94.6	**86.0**	80.2–90.3	65.9	49.3–79.4	90.8	81.4–95.9	**82.1**	73.6–88.3
A Disturbance of consciousness and attention	83.7 [73.5]	69.8–92.2	87.4 [88.7]	80.8–92.1	**86.5 [85.0]**	80.8–90.8	80.5 [68.3]	64.6–90.6	84.2 [88.2]	73.6–91.2	**82.9** **[81.2]**	74.6–89.0
B Cognition alteration or perceptual disturbance, not explained by a dementia.	83.7 [73.5]	69.8–92.2	80.1 [90.7]	72.7–86.0	**81.0 [86.5]**	74.7–86.0	80.5 [68.3]	64.6–90.6	78.9 [90.8]	67.8–87.1	**79.5 [82.9]**	70.8–86.2
C Acute onset and fluctuation tendency.	83.7 [71.4]	69.8–92.2	88.1 [89.4]	81.6–92.6	**87.0 [85.0]**	81.3–91.2	80.5 [65.9]	64.6–90.6	86.8 [88.2]	76.7–93.2	**84.6 [80.3]**	76.5–90.4
D Evidence for etiology.	75.5 [79.6]	60.8–86.2	81.5 [90.7]	74.1–87.1	**80.0 [88.0]**	73.6–85.2	70.7 [75.6]	54.3–83.3	80.3 [90.8]	69.2–88.2	**76.9 [85.5]**	68.0–84.0
DSM-5	73.5	58.7–84.6	88.1	81.6–92.6	**84.5**	78.6–89.1	68.3	51.8–81.4	86.8	76.7–93.2	**80.3**	71.8–86.9
A Disturbance in attention and awareness.	85.7 [73.5]	72.1–93.6	86.1 [88.1]	79.3–91.0	**86.0 [84.5]**	80.2–90.3	82.9 [68.3]	67.3–92.3	82.9 [86.8]	72.2–90.2	**82.9 [80.3]**	74.6–89.0
B Acute onset and fluctuation tendency.	83.7 [73.5]	69.8–92.2	88.1 [86.8]	81.6–92.6	**87.0 [83.5]**	81.3–91.2	80.5 [68.3]	64.6–90.6	86.8 [84.2]	76.7–93.2	**84.6 [78.6]**	76.5–90.4
C Additional cognitive change or perception disturbance.	98.0 [73.5]	87.8–99.9	36.4 [88.1]	28.9–44.7	**51.5 [84.5]**	44.4–58.6	97.6 [68.3]	85.6–99.9	14.5 [86.8]	7.8–24.8	43.6 **[80.3]**	34.5–53.1
D No better explanation by another neurocognitive disorder nor reduced level of arousal.	81.6 [75.5]	67.5–90.8	84.1 [88.1]	77.1–89.4	**83.5 [85.0]**	77.5–88.2	78.0 [70.7]	62.0–88.9	82.9 [86.8]	72.2–90.2	**81.2 [81.2]**	72.7–87.6
E Evidence for etiology.	75.5 [81.6]	60.8–86.2	81.5 [88.1]	74.1–87.1	**80.0 [86.5]**	73.6–85.2	70.7 [78.0]	54.3–83.3	80.3 [86.8]	69.2–88.2	**76.9 [83.8]**	68.0–84.0
ICD-10	53.1	38.4–67.2	96.0	91.2–98.4	**85.5**	79.7–89.9	48.8	33.1–64.6	93.4	84.7–97.5	**77.8**	**69.0–84.7**
A Clouding of consciousness and attention alteration.	81.6 [55.1]	67.5–90.8	90.1 [94.0]	83.9–94.1	**88.0 [84.5]**	82.5–92.0	78.0 [51.2]	62.0–88.9	88.2 [90.8]	78.2–94.1	**84.6 [76.9]**	76.5–90.4
B Disturbance of cognition (memory and orientation).	98.0 [55.1]	87.8–99.9	49.0 [96.0]	40.8–57.2	**61.0 [86.0]**	53.8–67.7	97.6 [51.2]	85.6–99.9	22.4 [93.4]	13.9–33.6	**48.7 [78.6]**	39.4–58.1
C One psychomotor disturbance (shifts from hypo to hyperactivity, reaction time increased, speech increased /decreased, enhanced startle reaction)	95.9 [53.1]	84.9–99.3	66.2 [96.0]	58.0–73.6	**73.5 [85.5]**	66.7–79.4	95.1 [48.8]	82.2–99.1	57.9 [93.4]	46.0–68.9	**70.9 [77.8]**	61.7–78.8
D Sleep-wake alteration (includes nocturnal worsening and hypnopompic disturbances)	71.4 [67.3]	56.5–83.0	72.2 [92.7]	64.2–79.0	**72.0 [86.5]**	65.1–78.0	70.7 [63.4]	54.3–83.3	61.8 [92.1]	49.9–72.5	**65.0 [82.1]**	55.5–73.4
E Rapid onset and fluctuations.	77.6 [53.1]	63.0–87.7	89.4 [95.4]	83.1–93.6	**86.5 [85.0]**	80.8–90.8	75.6 [48.8]	59.4–87.1	88.2 [92.1]	78.2–94.1	**83.8 [76.9]**	75.5–89.7
F Evidence for an etiologic cause.	77.6 [59.2]	63.0–87.7	80.1 [96.0]	72.7–86.0	**79.5 [87.0]**	73.1–84.7	73.2 [56.1]	56.8–85.2	76.3 [93.4]	64.9–85.0	**75.2 [80.3]**	66.2–82.5

Cluster analysis-defined groups were identified using DRS-R98 items. Performance characteristics and 95 % confidence intervals (95 % CI) are given for each classification system. Performance values for the diagnostic criteria after each individual criterion was excluded are noted within brackets. Bolded values denote when the percentage of correctly classified cases (accuracy) as compared to the reference standard are significant at *p* < 0.05 according to the Wald test

All diagnostic systems were relatively robust and, in general terms, maintained their classification performance when each individual criteria was excluded. Each of the individual criteria correctly classified subjects (*p* < 0.05), except for criterion C of DSM-III-R (57.5 %) and for criterion C of DSM-5 (43.6 %) in the demented subsample. DSM-5 criterion C had significant but low accuracy (51.5 %) in the whole sample. These two individual criteria were each compound (listing more than one type of symptom).

The cardinal criterion A from all diagnostic systems (attention) had high accuracies and reasonably well-balanced sensitivity and specificity. Evaluation of other cognitive symptoms obtained high sensitivity (98.0 % for ICD-10 and DSM-5), however specificity was very low (ICD-10 = 49.0 %; DSM-5 = 36.4 %). DSM-IV was better balanced (criterion B). Only DSM-III-R includes a criterion for disorganized thinking which performed well (89.8 % sensitivity, 79.5 % specificity). ICD-10 had criteria for psychomotor disturbance and sleep-wake cycle disturbance which performed moderately well.

As expected, Individual criteria with high sensitivity, as reported in Table 2, had the highest percentage of positivity for delirium within their corresponding whole sample or dementia subsample (containing Additional file 1: Table S1).

The results for the dementia subsample were similar to the whole sample except that accuracy, sensitivity and specificity were all slightly lower. The largest decrease in accuracy between the whole sample and the dementia subsample was for ICD-10 (from 85.5 % to 77.8 %). And when excluding an individual criterion, the largest reduction was for ICD-10 criterion evaluating memory and orientation (from 61.0 % to 48.7 %).

In the whole sample, the acute onset criteria (86.0–87.0 %) and the criteria including attentional disturbance (84.5–88.0 %) had the highest classification accuracy within each system. The highest individual criterion accuracy (88.0 %) was in ICD-10 for “clouding of consciousness and attention alteration.” This same pattern occurred in the dementia subsample though the values were slightly lower – 82.9–84.6 % and 80.3–84.6 %, respectively, with DSM-III-R performing the worst on each criterion.

### Reliability

Reliability of the four diagnostic systems is shown in Table 3. DSM-IV, DSM-III-R, and ICD-10 showed K values in the range of acceptable to good in the whole sample. DSM-5 did the best with the highest K value and when considering its individual criteria, also had most values in the good range irrespective of which sample was tested. In contrast, DSM-III-R performed the most poorly, with the highest number of questionable range K values in the dementia subsample. The reliability performance of both systems would remain almost the same if any of their individual criterion were excluded. No criterion performed in the unacceptable or excellent range.

**Table 3:** Reliability between two raters for delirium classification systems and their individual criteria

Classification Systems and their Criteria	Reliability whole sample (*n* = 200)		Reliability dementia subset (*n* = 117)	
	Kappa	95 % CI	Kappa	95 % CI
DSM-III-R	**0.62**	0.49–0.75	0.58	0.42–074
A Alteration to maintain and shift attention	**0.61** [0.58]	0.50–0.73	0.48 [0.52]	0.32–0.54
B Disorganized thinking	0.42 **[0.67]**	0.29–0.55	*0.35* **[0.61]**	0.18–0.52
C Alterations in two of: consciousness, perception, sleep – wake cycle, motor activity, orientation and memory.	**0.66 [0.61]**	0.55–0.77	*0.29* [0.57]	−0.02–0.60
D Acute onset and fluctuation tendency.	0.58 **[0.62]**	0.46–0.70	0.51 [0.58]	0.35–0.67
E Evidenced or presumed etiological cause.	0.45 **[0.62]**	0.33–0.57	*0.35* [0.58]	0.18–0.51
DSM-IV	**0.63**	0.50–0.76	0.54	0.37–0.72
A Disturbance of consciousness and attention	0.59 [0.56]	0.47–0.71	0.45 [0.47]	0.29–0.61
B Cognition alteration or perceptual disturbance, not explained by a dementia.	0.43 **[0.66]**	0.31–0.56	*0.31* [0.59]	0.15–0.48
C Acute onset and fluctuation tendency.	**0.61 [0.63]**	0.49–0.73	0.54 [0.53]	0.39–0.70
D Evidence for etiology.	0.57 **[0.64]**	0.46–0.68	0.47 [0.56]	0.31–0.62
DSM-5	**0.73**	0.62–0.84	**0.67**	0.53–0.82
A Disturbance in attention and awareness.	**0.67 [0.73]**	0.56–0.78	0.57 **[0.67]**	0.42–0.71
B Acute onset and fluctuation tendency.	**0.71 [0.71]**	0.60–0.81	**0.63 [0.65]**	0.48–0.77
C Additional cognitive change or perception disturbance.	0.47 **[0.73]**	0.33–0.62	*0.30* **[0.67]**	–0.5–0.64
D No better explanation by another neurocognitive disorder nor reduced level of arousal.	**0.68 [0.70]**	0.57–0.79	**0.61 [0.62]**	0.49–0.76
E Evidence for etiology.	0.58 **[0.72]**	0.47–0.69	0.46 **[0.65]**	0.31–0.61
ICD-10	0.57	0.42–0.73	0.49	0.29–0.68
A Clouding of consciousness and attention alteration.	0.58 [0.59]	0.45–0.70	0.45 [0.52]	0.29–0.61
B Disturbance of cognition (memory and orientation).	**0.69** [0.59]	0.59–0.79	0.52 [0.54]	0.32–0.72
C One psychomotor disturbance (shifts from hypo to hyperactivity, reaction time increased, speech increased /decreased, enhanced startle reaction)	0.52 [0.55]	0.40–0.64	0.49 [0.45]	0.32–0.65
D One alteration of sleep – wake (insomnia, nocturnal worsening, nightmares)	0.52 [0.56]	0.40–0.64	0.49 [0.52]	0.33–0.65
E Rapid onset and fluctuations.	0.58 [0.54]	0.45–0.71	0.50 [0.49]	0.34–0.66
F Evidence for an etiologic cause.	0.50 [0.57]	0.39–0.62	*0.37* [0.47]	0.21–0.53

Kappa for each classification system if each individual criterion were excluded is within brackets. Values in the questionable or unacceptable ranges are italicized. Values in the good range are bolded. K: <0.20 = unacceptable, 0.20–0.39 = questionable, 0.40–0.59 = acceptable, 0.60–0.79 = good, and 0.80–1 = excellent

Standard errors for each system and their individual criteria were all ≤0.1 with exception of the compound criterion C of DSM-III-R (SE 0.129) and the criterion C of DSM-5 for additional cognitive change/perception (SE 0.140) in the subset with dementia.

## Discussion

We describe a novel approach to evaluate how different delirium diagnostic systems perform in their ability to separate delirium and nondelirium groups, given that reliance on any particular diagnostic system a priori makes an assumption of superior validity if it is to be used as a reference standard. Instead, we applied cluster analysis of DRS-R98 items to a sample of 200 subjects to discern natural groups as the reference standard and then measured performance of four classification systems to diagnose delirium. The DRS-R98 uses phenomenological descriptive anchors for many delirium characteristics that were assessed in a standardized way, independently and without regard for a particular classification system (“agnostic”). Our DRS-R98 cluster analysis yielded two clearly differentiated groups, which indicates very good performance to serve as a reference standard. Additionally, dementia patients with or without delirium were included to increase diagnostic complexity.

Accuracy was very good for all diagnostic systems with DSM-III-R the highest (87.5 %) and DSM-5 the lowest (84.5 %). Overall, the classification performance in the dementia subsample was similar to but somewhat lower than in the whole sample, with ICD-10 performing the least well (77.8 %) and DSM-III-R somewhat better (83.8 %) than the other DSM versions. Values for sensitivity and specificity varied more than did accuracy in the whole sample, where the pattern for all was lower sensitivity than specificity. The most extreme was ICD-10 (53.1 %, 96.0 %) suggesting a better capacity for delirium confirmation, while the most balanced values were for DSM-III-R (81.6 %, 89.4 %). Each individual criterion, except one, significantly distinguished delirium and nondelirium groups in both the whole sample and dementia subsample.

Accuracies of diagnostic criteria remained robust even after each individual criterion was excluded such that they perform as an integrated whole. Exclusion of most of the individual criteria resulted in only small increases in classification accuracy of the remaining criteria. However, several individual criteria reduced overall classification accuracy before they were excluded and the most prominent of these had a compound construction (more than one type of symptom listed together). Inter-rater reliability for diagnostic systems was “good” except for ICD-10 that was “acceptable”, but none were excellent. ICD-10 had the lowest and DSM-5 had the highest interrater reliability.

The individual criteria across all classification systems with the highest accuracies were those for attentional disturbance and acute onset of symptoms, consistent with inattention being a cardinal feature and the syndrome being a noticeable change in consciousness. These might comprise the simplest screening approach for busy clinicians but has not been studied. Meagher at al. [8] reported that digit span forwards differentiated delirium from dementia subjects because simple inattention occurs in delirium more than in dementia, whereas both groups performed poorly on the more challenging backwards span test. A commonly used brief tool, the CAM [43], includes both inattention and acute onset among its four items, however, it does not have consistent concordance with DSM versions and DRS-R98 [6, 44].

These diagnostic systems varied greatly as to how many of the other cognitive, perceptual, thinking and circadian symptoms of delirium are represented. Interestingly the disorganized thinking criterion of DSM-IIIR performed well. However, the disorganized thinking was dropped as a criterion after DSM-III-R in order to improve the reliability of delirium diagnosis when assessed by non-psychiatrists [4]. However, as a core domain symptom our data suggest it should be included again in diagnostic criteria. Two other core domain symptoms, that describe circadian activity, have separate criteria in ICD-10 but performed only moderately well in accuracy. However they performed better than the “other cognitive” criterion in ICD-10.

None of these four diagnostic systems has individual criteria representing all three core domains of delirium (cognitive, circadian, and higher order thinking) [39–42]. DSM-III-R has disorganized thinking and ICD-10 has two circadian criteria. DSM-III-R includes more core domain symptoms than do the other DSM versions, though they are collapsed with “consciousness” into one compound criterion (i.e., consciousness, perception, sleep-wake cycle, motor activity, orientation and memory). This particular compound criterion was the only criterion from among all the systems whose accuracy was not significantly different between delirium and nondelirium groups. It would be worth studying new criteria that individually capture all three core domains.

Further, the compound criteria from DSM-III-R (C), DSM-IV (B), and DSM-5 (C) each carried lower accuracy contributions than when they were deleted. Because compound criteria, comprised of more than one type of symptom, had lower accuracies we recommend they be avoided in future diagnostic system revisions.

Accuracies were highest for the A criteria in each system, consistent with their being cardinal for the syndrome of delirium. Though other symptoms besides inattention had lower accuracies, such as evaluating other cognitive aspects, they showed high sensitivity despite low specificity. As such, they may be useful for delirium screening.

The wording of the cardinal A criterion varies across these systems, where DSM-IV and ICD-10 include mention “consciousness” along with inattention. Though contributing much to accuracy, interrater reliability was less strong when inattention was combined with consciousness as compared to cardinal criteria that only included the components of consciousness (i.e., attention and awareness). “Clouding of consciousness” has no precise or common definition however. Note that the DRS-R98 does not include vague items like “consciousness” or “clouding of consciousness.” Rather, the symptoms of delirium taken together should represent the components of an impairment of consciousness, where cerebral cortical arousal is intact (i.e., level of consciousness is not coma or stupor). Intact consciousness means being alert/attentive (and having other cognitive domains intact), awake (with an intact sleep-wake cycle), and aware (comprehending one’s inner self and one’s surroundings). So to include the term consciousness within the criteria is not helpful to delineate the particular features of delirium that would establish it as an impaired state of consciousness by its overall definition [44]. Thus, the raters would be influenced by their overall impression of the patient’s presentation during the interview to rate consciousness, similar to a clinical global impressions scale (CGI). DRS-R98 items do not include “consciousness” terms and can more cleanly establish the components of delirium when cluster analysis determined the groups. Because we found the highest accuracy (88.0 %) for the ICD-10 “clouding of consciousness and attention alteration” cardinal A criterion, it suggests that such wording functioned like a CGI rating and could be a candidate for a single screening question for use by clinicians in hospital settings.

Cognitive alterations are core for both dementia and delirium, and symptoms of the latter overshadow those of the former when they are comorbid [8, 21, 22], which may explain the decreased accuracy performance of diagnostic systems within the dementia subsample. Classification performance for all diagnostic systems in that subsample was slightly lower than in the whole sample, but over 80.0 % accuracy for all except ICD-10 that suffered the largest decline (7.7 percentage points). The ICD-10 criterion evaluating memory and orientation also had the highest accuracy drop within ICD-10 and among all individual criteria (12.3 percentage points) suggesting ICD-10 may not be as suitable for use in comorbid dementia cases though this needs confirmation in other studies.

Inter-rater reliability was highest for DSM-5 and, in the dementia subsample, the lowest for DSM-III-R when considering individual criteria reliabilities. Similar to a previous report of low ICD-10 reliability in general hospital inpatients, we found ICD-10 criteria had the worst reliability values [34]. Reliability values were somewhat lower in the dementia subsample overall as compared with the whole sample. As suggested by Regier et al. [1], comorbidity is usually associated with lower reliability values, especially when concurrent entities have shared symptoms, as happens with dementia and delirium. It could explain why although all diagnostic systems and individual criteria were very precise (95 % CI <0.5 and SE <0.1) in the whole sample, criteria that included cognitive aspects of delirium (criterion C in DSM-III-R and DSM-5) had SE a little over the desired 0.1 value in the subsample with dementia.

Though DSM-5 criteria had the best reliability, its accuracy in our sample was a little lower than the other systems, whereas DSM-III-R had the highest accuracy of 87.5 %. A previous report using latent class analysis found that DSM-III-R had higher accuracy than DSM-IV [5]. These findings, taken together, may be a consequence of the trend toward simplification of criteria over newer DSM editions which improve reliability at the expense of lowering accuracy. An alternative to oversimplification to enhance reliability for nonspecialists is to include operational descriptions for each criterion in future DSM versions, similar to what is available for the DRS-R98 Administration Guide (pdf available from Dr. Trzepacz at pttrzepacz@outlook.com).

Limitations include our use of only the DRS-R98 to capture characteristics of delirium. Designed for broad and detailed phenomenological descriptions of delirium features, it is ideal for this study’s purpose with advantages over other existing assessment tools that are not so structured. A reliable yet-to-be-determined biological marker, perhaps electroencephalography or fMRI, would be an important addition to phenotype criteria validity assessment, which we did not include.

## Conclusions

All diagnostic systems classified (>80.0 %) delirium from nondelirium cases as compared to an agnostic cluster-analysis reference standard, though all performed less well in the comorbid dementia subsample. The two best performing individual criteria across all classification systems were the attentional disturbance and acute onset features. Compound criteria (i.e., those with more than one type of symptom) tended to have lower accuracies and should be avoided in future diagnostic system revisions. None of the four diagnostic systems includes separate criteria that represent all three core domains of delirium (cognitive, circadian, higher order thinking).

In summary, ours is the first evaluation of four classification systems for delirium diagnosis that utilized comparisons of accuracy to an “agnostic” rating of symptoms using the DRS-R98 by an independent rater, and assessed classification performance characteristics of each system. This approach lends itself to discernment of how criteria are written in order to develop an even better set of diagnostic criteria that could truly serve as a reference standard.

## Abbreviations

CCI-SF, Charlson Comorbidity index, short form; CA, cluster analysis; CI, confidence interval; DRS-R98, delirium rating scale-revised-98; DSM, diagnostic and statistical manual of mental disorders; ICD, international classification of diseases; K, kappa index; MMSE, minimental state examination; S-IQCODE, spanish-informant questionnaire on cognitive decline in the elderly; SD, standard deviation; SE, standard error.

## Additional file

Additional file 1: Table S1. Frequency of patients positive for delirium according to each classification system and presence of their individual criteria, expressed for the whole sample (where the cluster analysis-defined delirium group was 49/200 patients or 24.5 %) and for the dementia subsample (where the cluster analysis-defined delirium group was 41/117, 35.0 %). (DOCX 15 kb)
