# Supplementary material for: Delirium diagnosis defined by cluster analysis of symptoms versus diagnosis by DSM and ICD criteria: diagnostic accuracy study
Source: BMC Psychiatry. 2016 May 26;16:167. doi: 10.1186/s12888-016-0878-6 (PMC4882791; doi:10.1186/s12888-016-0878-6)
Supplement: Additional file 1: Table S1. — Frequency of patients positive for delirium according to each classification system and presence of their individual criteria, expressed for the whole sample (where the cluster analysis-defined delirium group was 49/200 patients or 24.5 %) and for the dementia subsample (where the cluster analysis-defined delirium group was 41/117, 35.0 %). (DOCX 15 kb) [file 12888_2016_878_MOESM1_ESM.docx]

**Additional file 1: Table S1.** Frequency of patients positive for delirium according to each classification system and presence of their individual criteria, expressed for the whole sample (where the cluster analysis-defined delirium group was 49/200 patients or 24.5%) and for the dementia subsample (where the cluster analysis-defined delirium group was 41/117, 35.0%).

| **Diagnostic Criteria** | **Whole sample**  **(n =200)** | **Dementia subsample**  **(n =117)** |
| --- | --- | --- |
|  | **n (%)** | **n (%)** |
| **DSM-III-R** | 56 (29.5) | 42 (35.9) |
| **A** Alteration to maintain and shift attention | 66 (33.0) | 50 (42.7) |
| **B** Disorganized thinking | 75 (37.5) | 60 (51.3) |
| **C** Alterations in two of: consciousness, perception, sleep-wake cycle, motor activity, orientation and memory. | 134 (67.0) | 103 (88.0) |
| **D** Acute onset and fluctuation tendency. | 59 (29.5) | 43 (36.7) |
| **E** Evidenced or presumed etiological cause. | 70 (35.0) | 49 (41.9) |
| **DSM-IV** | 49 (24.5) | 34 (29.0) |
| **A** Disturbance of consciousness and attention | 60 (30.0) | 45 (38.5) |
| **B** Cognition alteration or perceptual disturbance, not explained by a dementia. | 71 (35.5) | 49 (41.9) |
| **C** Acute onset and fluctuation tendency. | 59 (29.5) | 43 (36.7) |
| **D** Evidence for etiology. | 65 (32.5) | 44 (37.6) |
| **DSM-5** | 54 (27.0) | 38 (32.5) |
| **A** Disturbance in attention and awareness. | 63 (31.5) | 47 (40.2) |
| **B** Acute onset and fluctuation tendency. | 59 (29.5) | 43 (36.7) |
| **C** Additional cognitive change or perception disturbance. | 144 (72.0) | 105 (89.7) |
| **D** No better explanation by another neurocognitive disorder nor reduced level of arousal. | 64 (32.0) | 45 (38.5) |
| **E** Evidence for etiology. | 65 (32.5) | 44 (37.6) |
| **ICD-10** | 32 (16.0) | 25 (21.4) |
| **A** Clouding of consciousness and attention alteration. | 55 (27.5) | 41 (35.0) |
| **B** Disturbance of cognition (memory and orientation). | 125 (62.5) | 99 (84.6) |
| **C** One psychomotor disturbance (shifts from hypo to hyperactivity, reaction time increased, speech increased /decreased, enhanced startle reaction) | 98 (49.0) | 71 (60.7) |
| **D** Sleep-wake alteration (includes nocturnal worsening and hypnopompic disturbances) | 77 (38.5) | 58 (49.6) |
| **E** Rapid onset and fluctuations. | 54 (27.0) | 40 (34.2) |
| **F** Evidence for an etiologic cause. | 68 (34.0) | 48 (41.0) |
